# Altered expression of neuropeptide Y receptors caused by focal cortical dysplasia in human intractable epilepsy

**DOI:** 10.18632/oncotarget.7855

**Published:** 2016-03-02

**Authors:** Lin Li, Jiahui Deng, Changqing Liu, Hanjiang Luo, Yuguang Guan, Jian Zhou, Xueling Qi, Tianfu Li, Zhiqing David Xu, Guo-Ming Luan

**Affiliations:** ^1^ Department of Functional Neurosurgery, Sanbo Brain Hospital, Capital Medical University, Beijing, P.R. China; ^2^ Beijing Key Laboratory in Epilepsy, Beijing, P.R. China; ^3^ Center of Epilepsy, Beijing Institute for Brain Disorders, Beijing, P.R. China; ^4^ Beijing Key Laboratory of Neural Regeneration and Repair, Department of Neurobiology, Beijing Institute for Brain Disorders, Capital Medical University, Beijing, P.R. China; ^5^ Department of Neuroscience, Karolinska Institute, Stockholm, Sweden

**Keywords:** focal cortical dysplasia, neuropeptide Y Y1 receptor, neuropeptide Y Y2 receptor, neuropeptide Y Y5 receptor, Pathology section

## Abstract

Focal cortical dysplasia (FCD) is a common cause of pharmacologically-intractable epilepsy, however, the precise mechanisms underlying the epileptogenicity of FCD remains to be determined. Neuropeptide Y (NPY), an endogenous anticonvulsant in the central nervous system, plays an important role in the regulation of neuronal excitability. Increased expression of NPY and its receptors has been identified in the hippocampus of patients with mesial temporal lobe epilepsy, presumed to act as an endogenous anticonvulsant mechanism. Therefore, we investigated whether expression changes in NPY receptors occurs in patients with FCD. We specifically investigated the expression of seizure-related NPY receptor subtypes Y1, Y2, and Y5 in patients with FCD versus autopsy controls. We found that Y1R and Y2R were up-regulated at the mRNA and protein levels in the temporal and frontal lobes in FCD lesions. By contrast, there was no significant change in either receptor detected in parietal lesions. Notably, overexpression of Y5R was consistently observed in all FCD lesions. Our results demonstrate the altered expression of Y1R, Y2R and Y5R occurs in FCD lesions within the temporal, frontal and parietal lobe. Abnormal NPY receptor subtype expression may be associated with the onset and progression of epileptic activity and may act as a therapeutic candidate for the treatment of refractory epilepsy caused by FCD.

## INTRODUCTION

Focal cortical dysplasia (FCD) is a malformation of cortical development commonly associated with medically refractory epilepsy. In 1971, Taylor *et al.,* first described focal anomalies of cortical structure [1]. FCD has since been described as a distinct pathological substrate, characterized by disruption of the cortical laminar architecture and/or the appearance of abnormal cells [2]. Although the exact etiology of epileptogenicity in FCD remains unknown, it is believed that the imbalance between inhibitory and excitatory neuronal circuits may contribute to the initiation and progression of epileptic seizures [3]. Specifically, altered number and function of inhibitory interneurons is proposed as a mechanism leading to an FCD imbalance [4, 5]. Neuropeptide Y (NPY) is expressed in subpopulations of interneurons and acts on G-protein coupled receptors to exert a powerful effect on pre- and post-synaptic transmission. NPY has been proposed as an ideal modulator of seizures given its localization within dense core vesicles and release during sustained high-frequency stimulation [6]. Indeed, increasing evidence indicates that NPY is a powerful endogenous regulator of limbic seizure activity [7-9]. Changes in NPY system have been shown both in animal models and patients with mesial temporal lobe epilepsy (mTLE). Seizure-induced upregulation in the synthesis and release of NPY as well as altered expression of NPY receptors in hippocampus are generally believed to represent a compensatory anticonvulsant response. In keeping with this hypothesis, Thom *et al*. [4] reported a striking increase in the density of NPY immunoreactive fibers plexus in FCD, which may also represent an adaptive anti-epileptic mechanism to dampen down seizure propagation. However, it still remains unclear which NPY receptor subtypes are responsible for mediating the anticonvulsant effect of NPY in the neocortex. *In vivo* and *in vitro* studies using various antagonists and agonists for NPY receptors have reported conflicting results, further complicating our understanding of this process [10]. In the present study, to further elucidate the mechanism underlying the protective role of NPY in the human neocortex, we investigated the expression of Y1, Y2 and Y5 receptors in FCD specimens obtained from patients with refractory epilepsy during surgery and in postmortem tissue from non-neurological disease patients. Our data provided a basic understanding of the plasticity of NPY receptors in patients with FCD.

## RESULTS

### Characterization and distribution of Y1R

**Figure 1:**
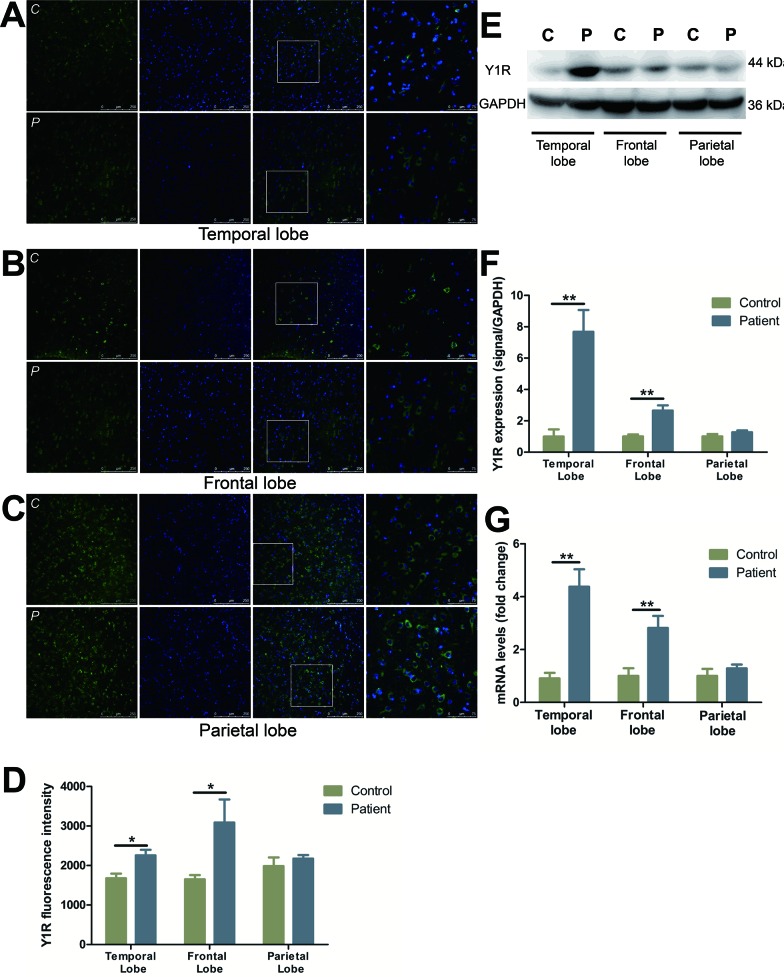
The expression and distribution of Y1R in different cortical lobes of FCD patients **A.**-**C.** Representative images show Y1R (green) and DAPI (blue) in temporal lobe (A), frontal lobe (B) and parietal lobe (C) with upper panels showing control and lower panels showing patients. The last images are the enlarged views from areas indicated by squares. C, control; P, patient. **D.** The quantification of Y1R fluorescent intensities of A-C. **E.** Representative Western blots of Y1R proteins in the temporal, frontal and parietal lobe. **F.** Bar graphs show quantitative data for Y1R signals that are normalized to GAPDH signal. **G.** Quantitative PCR array analysis of the expression of Y1R in the temporal, frontal and parietal lobe. Data are expressed by means ± SEM. *P < 0.05, **P < 0.01, ***P < 0.001 compared with control group.

Y1R immunostaining showed a marked increase in neuronal cells in temporal (by 34.4%) (Figure 1A, 1D) and frontal (by 87.0%) (Figure 1B, 1D) lobes, as compared to control subjects. No changes, however, were observed in parietal lobe (Figure 1C, 1D). Western blot analysis demonstrated Y1R expression was elevated in the temporal and frontal lobes of FCD patients as compared to controls (Figure 1E, 1F). In accordance with the western blot studies, the mRNA levels of Y1R were detected 4.8- and 2.8- fold higher in the temporal and frontal lobes, respectively, of FCD patients as compared to controls (Figure 1G). However, there was no significant change in the mRNA or protein expression in parietal lobe between FCD and controls (Figure 1C-1G).

### Characterization and distribution of Y2R

**Figure 2:**
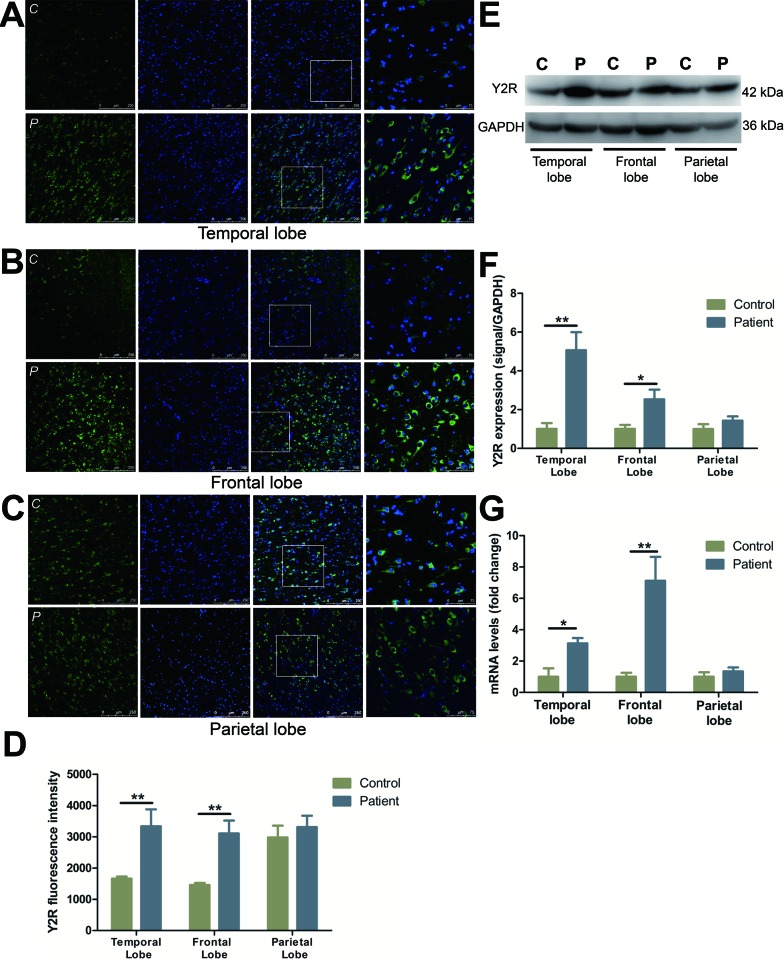
The expression and distribution of Y2R in different cortical lobes of FCD patients **A.**-**C.** Immunofluorescent images illustrate Y2R (green) and DAPI (blue) in temporal lobe (A), frontal lobe (B) and parietal lobe (C) with upper panels showing control and lower panels showing patients. The last images are the enlarged views from areas indicated by squares. C, control; P, patient. **D.** The quantification of Y2R fluorescent intensities of A-C. **E.** Representative Western blots of Y2R proteins in the temporal, frontal and parietal lobe. **F.** Bar graphs show quantitative data for Y2R signals that are normalized to GAPDH signal. **G.** Quantitative PCR array analysis of the expression of Y2R in the temporal, frontal and parietal lobe. Data are expressed by means ± SEM. *P < 0.05, **P < 0.01, compared with control group.

Y2R immunoreactive staining was increased in temporal (by 101.1%) (Figure 2A, 2D) and frontal lobes (by 113.2%) (Figure 2B, 2D) as compared to the controls. Y2R in normal parietal lobe was expressed at a higher level of fluorescence intensity than other normal cortical regions, but displayed little change compared to FCD patients (Figure 2C, 2D). Y2R protein expression was 406.2% and 153.2% increased in the temporal and frontal lobes in FCD patients *versus* controls (Figure 2E, 2F). A similar trend was observed at the mRNA level, with a 7.1- and 3.1-fold increase in Y2R expression in the frontal lobe and temporal lobe, respectively. No significant differences in the mRNA expression of Y2R was observed in the parietal lobe (Figure 2G).

### Characterization and distribution of Y5R

**Figure 3:**
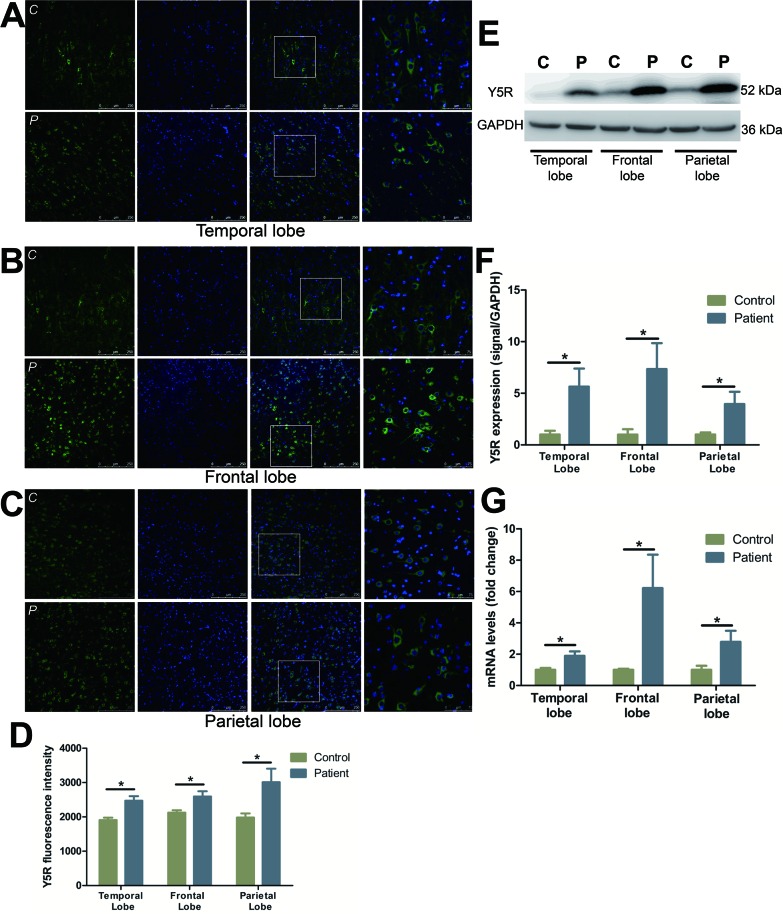
The expression and distribution of Y5R in different cortical lobes of FCD patients **A.**-**C.** Representative images show Y5R (green) and DAPI (blue) in temporal lobe (A), frontal lobe (B) and parietal lobe (C) of control (upper panels) and patients (lower panels). The last images are the enlarged views from areas indicated by squares. C, control; P, patient. **D.** The quantification of Y5R fluorescent intensities of A-C. **E.** Representative Western blots of Y5R proteins in the temporal, frontal and parietal lobe. **F.** Bar graphs show quantitative data for Y5R signals that are normalized to GAPDH signal. **G.** Quantitative PCR array analysis of the expression of Y5R in the temporal, frontal and parietal lobe. Data are expressed by means ± SEM. *P < 0.05 compared with control group.

Y5R staining in FCD patients demonstrated a significant increase in the number of positive cells, particularly those localized within the parietal lobe (Figure 3A-3D) (temporal lobe: by 29.4%; frontal lobe: by 22.1%; parietal lobe: by 51.9%). The expression of Y5R in all three lobes increased notably in FCD patients as compared to the controls in the temporal (by 464.8%), frontal (by 633.9%) and parietal lobes (by 295.5%), respectively (Figure 3E, 3F). Y5R mRNA levels similarly demonstrated a significant increase in expression with a 1.9-, 6.2-, and 2.8-fold change in comparison to the controls in temporal, frontal and parietal lobes, respectively (Figure 3G).

### Characterization and distribution of NPY

**Figure 4:**
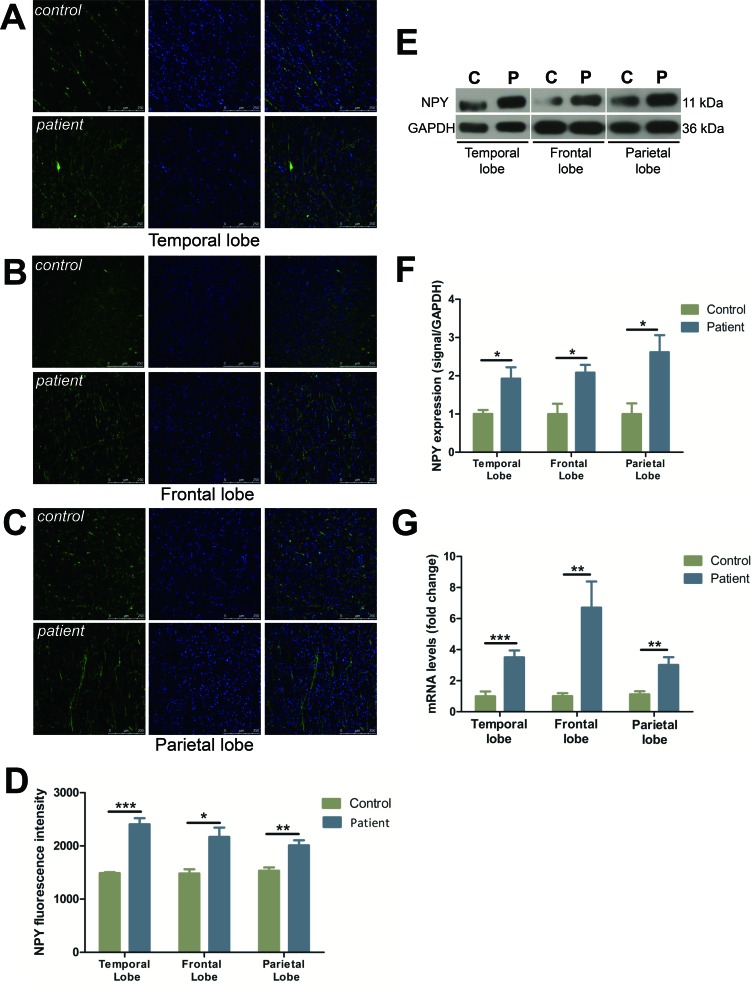
The expression of NPY was increased in different cortical lobes of FCD patients **A.**-**C.** Immunofluorescent images show NPY (green) and DAPI (blue) in temporal lobe (A), frontal lobe (B) and parietal lobe (C) with upper panels showing controls and lower panels showing patients. C: control; P: patient. **D.** The quantification of NPY fluorescent intensities of A-C. **E.** Representative Western blots show NPY protein expression in the temporal, frontal and parietal lobe. **F.** Bar graphs show quantitative data for NPY signals that are normalized to GAPDH signal. **G.** Quantitative PCR array analysis of the expression of NPY in the temporal, frontal and parietal lobe. Data are expressed by means ± SEM. *P < 0.05, **P < 0.01, ***P < 0.001 compared with control group.

FCD cases all demonstrated a striking increase in the density of the NPY fiber plexus as compared to the controls in temporal (by 61.4%), frontal (by 46.3%), and parietal lobes (by 31.2%), respectively (Figure 4A-4D). Western blot analysis demonstrated NPY expression was elevated in all FCD resections as compared to controls (temporal lobe: by 92.5%, frontal lobe: by 108.1%, parietal lobe: by 161.4%) (Figure 4E, 4F). NPY mRNA levels demonstrated a significant increase in expression with a 3.5-, 6.7-, and 3.0-fold change in comparison to the controls in temporal, frontal and parietal lobes, respectively (Figure 4G).

## DISCUSSION

The main objective of this study was to explore expression patterns of NPY receptor subtypes (Y1, Y2 and Y5) in refractory epilepsy caused by FCD. We found that Y1R and Y2R were up-regulated at both the mRNA and protein levels in both the temporal and frontal lobes in FCD. By contrast, there was no significant change in expression of these receptors in parietal lesions. Notably, overexpression of Y5R, at the mRNA or protein level, was observed in all cortical resections.

NPY is considered to be a powerful endogenous anticonvulsant [11-13]. Elevated expression of NPY has been identified in the brains of patients with FCD [4]. In keeping with previously published results, we detected a marked increase in the density of the NPY fiber plexus in FCD resections (Figure 4A-4D), which may represent an endogenous anticonvulsant mechanism. Previous reports have suggested that seizure-induced altered expression of NPY is accompanied by changes in NPY receptor subtypes [14, 15]. We focused our analysis on Y1R, Y2R and Y5R due to their abundant expression in the human brain and close relationship to epileptogenesis.

Since their discovery, Y1R and Y2R have received significant attention, however, conflicting reports have limited our ability to delineate their specific roles in seizure. Increasing evidence has suggested the anticonvulsant effect of NPY in the hippocampus and neocortex is mediated through different NPY receptor subtypes [16, 17]. In the hippocampus, Y2R has generally been viewed to play a critical role in the anticonvulsant properties of NPY. Hippocampal Y2Rs are observed at relatively high levels in terminal regions of Schaffer collaterals and mossy fibers [18], and the presynaptic location, which fits with the reported inhibitory effect of Y2R on voltage-dependent Ca^2+^-mediated glutamate release and synaptic transmission. Comers *et al.,* reported that NPY suppresses hippocampal epileptiform activity through the activation of Y2R [17, 19]. This effect, however, can be blocked using a selective Y2R antagonist in rat hippocampal slice [20]. Furthermore, it has been reported that mossy fiber sprouting is associated with an increased expression of Y2R following recurrent seizures in mTLE patients and epileptic rats [21-23]. Taken together, these results suggest Y2R participates in NPY antiepileptic activity in hippocampus. By contrast, in the neocortex NPY can induce long-lasting increases in GABAergic neurotransmission on pyramidal cells suppressing the excitability in cortical circuits [24]. Y1R is highly expressed in the neocortex, while Y2R is observed at much less prominent levels [25-28]. The predominant postsynaptic expression of Y1R suggests it may be the primary effector of NPY-induced changes in the neocortex. However, scarce information is available on the role of Y1R and Y2R within this region of the brain. In the present study, Y1Rs and Y2Rs were significantly up-regulated in both temporal and frontal FCD lesions (Figure 1A, 1B, and 1D-1E; Figure 2A, 2B, and 2D-2E). It is believed that a disbalance of inhibitory and excitatory neurotransmission contributed to the intrinsic epileptogenicity of FCD. Our results demonstrated that internal NPY receptor system may work to counteract to this disbalance in both temporal and frontal lobes by up-regulating Y1R and Y2R expression. Owing to the low expression of Y2R in cortex, we speculate that the anticonvulsant effect of NPY is predominantly mediated by Y1R in FCD lesions.

It is noteworthy that no change in Y1R and Y2R expression was detected in parietal resections in either the mRNA or protein levels (Figure 1C-1G, Figure 2C-2G). We hypothesize that NPY receptors are associated with numerous physiological and pathological processes within the central nervous system (CNS), including sleep regulation, food intake, tissue growth, stress response and inflammatory processes [29], and any disruption of these processes can contribute to the alteration of the NPY system. As epileptic patients often suffer from CNS disorders including anxiety and depression [30, 31], among others, it should be noted that abnormal expression of NPY receptors may be the consequence of an integrated response of NPY system to comorbidity.

Although the NPY anti-convulsant effect seems to be predominantly mediated through Y1R and/or Y2R, several lines of evidence have indicated that activation of Y5R can exert seizure-suppressant effect in extra-hippocampal regions [32]. Within the hippocampus, Y5R overexpression by itself has no anticonvulsant effect, while combined overexpression of Y5R and NPY exerts a pronounced inhibitory effect on seizures [33]. Moreover, transgenic studies have shown that mice lacking Y5R result in a more sever systematic kainate seizures, as compared to wildtype mice [32, 34]. Taken together, these findings suggest Y5R modulates NPY's antiepileptic activity. In the present study, we observed a robust increase in Y5R expression, at both the mRNA and protein levels, in all FCD lesions (Figure 3). Very little is known about the effect of Y5R in human neocortex. We speculates that overexpression of Y5Rs, combined with NPY upregulation, can suppress seizure activity effectively in FCD lesions. Since Y5R resides on postsynaptic neurons [35], the inhibitory nature of Y5R may be similar as Y1R. Our findings suggest that Y5R is a component of NPY action in the human neocortex.

NPY is well known for its ability to suppress seizures. From a clinical viewpoint, NPY is an attractive target for the development of next-generation antiepileptic drugs (AEDs) [36]. In the present study, we characterized the plasticity of seizure-associated NPY receptor subtypes within the regionally selective epileptic focus to provide insight into the mechanisms underlying the anticonvulsant action of NPY in the neocortex. It is worth noting that our data is restricted to the quantitative alteration of NPY receptors, and further receptor binding and functional experiments are needed to completely characterize our understanding of these receptors.

In summary, this study illustrates the changes in expression of three NPY receptor subtypes in the context of FCD. Our data suggests up-regulation of Y1R, Y2R and Y5R may play an important role in the seizure activity of refractory epilepsy caused by FCD.

## MATERIALS AND METHODS

### Patient cohort

This study was approved by the Institutional Review Board of the Sanbo brain hospital. We retrospectively reviewed clinical data of 32 FCD patients with medically refractory epilepsy who were surgically treated at SanBo Brain Hospital. The patients provided informed consent for both clinical data collection and the examination of surgical specimens. The clinical parameters of the cohort are summarized in Table 1. All patients received comprehensive presurgical evaluation. The extent of resection was preoperatively determined using information obtained from neurophysiology and neuroradiology. Preoperative brain MRI was performed as part of inpatient evaluation for all candidates and demonstrated the presence of a lesion in all patients. All patients underwent scalp video-EEG to determine the location of seizure onset. At least 3 seizures were recorded whose clinical semiology and epileptiform discharges were consistent with an origin at the anatomical location of the lesion. The pathological report for FCD was available for all patients. The diagnosis was confirmed by three pathologists, according to the updated FCD classification scheme [37].

**Table 1:** Clinical data of patients with FCD

Localization	Number of patients	Pathological type			Mean age at surgery (range)(year)	Mean duration of epilepsy (range)(year)
		FCDI	FCDII	FCDIII		
Temporal lobe	12	8	1	3	22.3±7.7 (12-41)	12.8±9.9 (2-33)
Frontal lobe	9		9		11.0±6.3 (2-20)	6.7±6.2 (1-19)
Parietal lobe	11		11		11.7±6.8 (3-26)	8.0±6.2 (2-22)

FCD: Focal Cortical Dysplasia. Data are expressed by means ± SD.

Normal cortex was obtained at routine autopsy. The cases had no known history of neurological or psychiatric disease, and each brain was studied by three pathologists to confirm the absence of any brain lesion. The clinical parameters of autopsy subjects are summarized in Table 2.

**Table 2:** Information of normal subjects

Subject	Age (years)/gender	PMI (h)	Cause of death
A	56/M	10	liver cirrhosis
B	64/F	20	pneumonia
C	47/F	12	renal failure
D	37/M	7	myocardial infarction
E	40/M	6	myocardial infarction

PMI: Post mortem interval. M: Male F: Female

The surgical samples (FCD and normal tissues) used for western blot and RT-PCR were snap-frozen immediately following resection and were stored at −80°C. Specimens used for immunohistochemistry were fixed in neutral buffered formalin and processed in paraffin.

### Real-time (RT)-PCR

RNA was extracted from brain tissue using RNeasy Lipid Tissue Mini Kit (Qiagen). Five micrograms (5μg) of total mRNA was reverse transcribed using Superscript III First-Strand Synthesis System for RT-PCR (Invitrogen). Quantitative PCR was performed using SYBR Green Mix (ABI). The polymerase chain reaction (PCR) cycling parameters were 50°C for 2 min, 95°C for 10 min, 40 cycles of 95°C for 15 s, and 60°C for 1 min. GAPDH was used as a housekeeping control gene. The primer sequences were as follows: Y1R forward CTG GAA ACC TGG CCT TGA T, Y1R reverse GAT GGC AAC AAG CAA GTC TG, Y2R forward TGA TGA GAA CCA GAC AGT GGA, Y2R reverse CAA GCA AGA TGA TGG AGC AG, Y5R forward TTG TCA TTT GTT GGG CAT GA, Y5R reverse CAG CAT TTG TTC TTT CCT TGG, NPY forward GCT GCG ACA CTA CAT CAA CC, NPY reverse ATT TCC CAT CAC CAC ATT GC, and GAPDH forward GAC CAC CCA GCC CAG CAA GG, GAPDH reverse TCC CCA GGC CCC TCC TGT TG. All reactions performed in triplicate.

### Western blots

Tissues were lysed in 2% SDS buffer, and protein concentration was determined using the BCA assay kit (Pierce). Equal amounts of protein (50μg) were separated on 10% SDS-PAGE gels and transferred to polyvinylidene difluoride membranes (Millipore). Antibodies against Y1R (1:200, Alomone Labs), Y2R (1:200, Alomone Labs), Y5R (1:200, Alomone Labs), NPY (1:200, Sigma), and GAPDH (1:5000, Sigma) were used in this study. The membranes were treated with anti-rabbit or anti-mouse horseradish peroxidase conjugated antibody (1:10,000; Amersham) for 1 hour and detected by ECL chemiluminescence substrate (Amersham). Band intensities were measured with the Image J program.

### Immunohistochemistry (IHC)

Five micron (5μm) paraffin-embedded brain sections were immersed in 0.01 M sodium citrate buffer (pH 6.0) for high-temperature heating antigen retrieval. The sections were incubated with antibodies against Y1R (1:200, Alomone Labs), Y2R (1:200, Alomone Labs), Y5R (1:200, Alomone Labs), NPY (1:200, Sigma). TSA plus TMR/Fluorescence kit (PerkinElmer) was used to visualize the antigen-antibody reaction. Nuclei were counterstained with DAPI (1:1000, Invitrogen). Images were captured using a laser scanning confocal microscope (Leica TCS SP8, Germany).

### Statistical analysis

Data is represented as either means ± SD, or means ± SEM and analyzed by an unpaired Student's *t*-test as appropriate by using Prism 5.0 software (GraphPad Software, San Diego, CA). A p-value of less than 0.05 (*P* < 0.05) was considered a statistically significant.
